# Usefulness of phase gradients of otoacoustic emissions in auditory health screening: An exploration with swept tones

**DOI:** 10.3389/fnins.2022.1018916

**Published:** 2022-10-17

**Authors:** Xin Wang, Mingxing Zhu, Yuchao He, Zhenzhen Liu, Xin Huang, Hongguang Pan, Mingjiang Wang, Shixiong Chen, Yuan Tao, Guanglin Li

**Affiliations:** ^1^CAS Key Laboratory of Human-Machine Intelligence-Synergy Systems, Shenzhen Institute of Advanced Technology, Chinese Academy of Sciences, Shenzhen, China; ^2^Shenzhen College of Advanced Technology, University of Chinese Academy of Sciences, Shenzhen, China; ^3^Guangdong-Hong Kong-Macao Joint Laboratory of Human-Machine Intelligence-Synergy Systems, Shenzhen Institute of Advanced Technology, Chinese Academy of Sciences, Shenzhen, China; ^4^School of Electronics and Information Engineering, Harbin Institute of Technology, Shenzhen, China; ^5^Surgery Division, Epilepsy Center, Shenzhen Children’s Hospital, Shenzhen, China; ^6^Department of Otorhinolaryngology, Peking University Shenzhen Hospital, Shenzhen, China; ^7^Department of Otolaryngology, Shenzhen Children’s Hospital, Shenzhen, China

**Keywords:** swept-tone, otoacoustic emissions, cochlear impairment, phase gradients, auditory health screening

## Abstract

Otoacoustic emissions (OAEs) are low-level sounds generated by the cochlea and widely used as a noninvasive tool to inspect cochlear impairments. However, only the amplitude information of OAE signals is used in current clinical tests, while the OAE phase containing important information about cochlear functions is commonly discarded, due to the insufficient frequency-resolution of existing OAE tests. In this study, swept tones with time-varying frequencies were used to measure stimulus frequency OAEs (SFOAEs) in human subjects, so that high-resolution phase spectra that are not available in existing OAE tests could be obtained and analyzed. The results showed that the phase of swept-tone SFOAEs demonstrated steep gradients as the frequency increased in human subjects with normal hearing. The steep phase gradients were sensitive to auditory functional abnormality caused by cochlear damage and stimulus artifacts introduced by system distortions. At low stimulus levels, the group delays derived from the phase gradients decreased from around 8.5 to 3 ms as the frequency increased from 1 to 10 kHz for subjects with normal hearing, and the pattern of group-delay versus frequency function showed significant difference for subjects with hearing loss. By using the swept-tone technology, the study suggests that the OAE phase gradients could provide highly sensitive information about the cochlear functions and therefore should be integrated into the conventional methods to improve the reliability of auditory health screening.

## Introduction

Otoacoustic emissions (OAEs) are low-level sounds generated by the normal activities of the cochlea and can be recorded by a sensitive microphone inside the ear canal. Although it is difficult to physically examine the cochlea due to its deep location inside the temporal bone, the discovery of OAEs provides a non-invasive window to observe the functional status of the cochlea. Studies have shown that OAEs are byproducts of the mechano-electrical activities of outer hair cells (OHCs) that can provide energy feedback to boost the vibrations of the basilar membrane and to amplify the cochlear response to incoming sounds (Dallos et al., 1997). Such physiological OHC activities, also called electromotility, are crucial for the extraordinary frequency selectivity and hearing sensitivity of the human auditory system (Liberman et al., 2002). During the active process of OHC electromotility, part of the extra energy provided by OHCs travels inversely along the basilar membrane and propagates to the ear canal and is recorded as OAEs. A study showed that OAE signals would significantly decrease or even disappear as a result of the blockage of the OHC electromotility (Brownell, 1990). Therefore, the presence of OAEs is a reliable indicator of thriving OHC activities as well as a normal functioning cochlea. Moreover, OAE signals are easy to measure and not affected by attentions or consciousness of the patients (Meric and Collet, 1994). Therefore, OAE measurements have been intensely used in routine hearing screening and audiological assessments in the clinic, especially for the pediatric population that is difficult to test in conventional audiogram assessments (Kemp et al., 1990).

Otoacoustic emission signals are commonly analyzed in the frequency domain by examining both amplitude and phase spectra. Since OHCs at different cochlear positions generate OAEs of different frequencies, their amplitude spectrum has been widely used as a direct approach to inspect the presence of OAE signals and to evaluate OHC functionalities at different frequencies. The OAE amplitude spectrum demonstrates regular spectral periodicity that is unique to each subject (Neumann et al., 1994; Talmadge et al., 1999; Wagner et al., 2008). Another distinctive feature of OAE signals is that the phase changes dramatically with frequency and there is a steep phase gradient in the OAE phase spectrum (Dhar et al., 2002; Choi et al., 2008; Martin et al., 2009; Henin et al., 2011). The steep phase gradient, which reflects the round-trip travel time of OAE signals, is closely related to the active process of OHC electromotility and the frequency selectivity of the cochlea (Shera and Bergevin, 2012). The unique feature of phase gradient enables it to act as an important tool for various studies in the peripheral auditory system (Shera and Guinan, 1999, 2003; Kalluri and Shera, 2001). The phase gradient could also be utilized to investigate the sources and generation mechanisms of different types of OAEs (Zweig and Shera, 1995; Shera and Guinan, 1999; Kalluri and Shera, 2001; Goodman et al., 2003; Lineton and Lutman, 2003), to estimate the cochlear tuning by deducting group delays of the basilar membrane at different frequencies (Shera et al., 2002; Siegel et al., 2005; Shera and Bergevin, 2012), and to examine the olivocochlear efferent control of OHC activities introduced by a contralateral stimulus (Giraud et al., 1995; Guinan et al., 2003). Moreover, a recent study showed that there was a close relation between unstable OAE phase shift and a stiff cochlear partition, suggesting that the phase could be possibly used as a non-invasive way to detect endolymphatic hydrops of Menière’s disease (Avan et al., 2011).

Although the OAE phase gradients are useful in different ways, they are mostly restricted to auditory research only and rarely used in clinical practices (Abdala et al., 2018; Liu et al., 2020). Currently, two types of OAEs are measured in the clinics: transient evoked otoacoustic emissions (TEOAEs) measured with brief tones such as clicks, and distortion product otoacoustic emissions (DPOAEs) induced by two sinusoids with closely spaced frequencies. However, only the OAE amplitude is provided upon the completion of both types of measurements while the phase information which essentially represents the OAE signals is completely discarded. One reason is that the measure of phase information requires that the OAEs be tested with a sufficient frequency resolution so that the phase difference between two neighboring frequencies does not exceed 2π to avoid possible phase discontinuities (Shera et al., 2002). However, the frequency resolution of current OAE measurements is usually in the order of hundreds of hertz and it is far from sufficient to capture OAE phases that change dramatically with frequency (Shera and Bergevin, 2012). Recently, Chen et al. (Chen et al., 2013; Jun et al., 2014) proposed a method of using swept tones with rapidly varying frequencies to measure OAE signals with a frequency-resolution as high as a few hertz, making it a great candidate to measure the phase of OAE signals across frequencies. Another possible reason for the lack of use of OAE phases is that the phases of TEOAEs and DPOAEs could be deteriorated by their complex generation mechanisms (Shera and Guinan, 1999). The multiple reflections of TEOAEs (Kemp et al., 1990; Avan et al., 1993; Tognola et al., 1997) and two distinctive sources of DPOAEs (Shera and Guinan, 1999, 2003; Kalluri and Shera, 2001) make it rather difficult to interpret the phase information of the two types of OAEs currently used in the clinic. Stimulus frequency otoacoustic emission (SFOAE) is another type of OAEs commonly evoked by one single stimulus and it attracts increasing attention recently due to its appealing features when compared with other types of OAEs (Guinan et al., 2003; Choi et al., 2008; Bentsen et al., 2011; Cheatham et al., 2011; Ren et al., 2013). Studies showed that SFOAEs were more frequency-specific in reflecting the functional status of corresponding OHCs (Guinan et al., 2003; Choi et al., 2008). It is widely accepted that SFOAEs are generated by linear coherent reflections within the cochlea (Shera and Guinan, 1999; Goodman et al., 2003; Lineton and Lutman, 2003), and therefore the interpretation of SFOAEs is less complicated, by avoiding multiple reflections in TEOAEs and source mixing in DPOAEs.

The purpose of this study is to use the swept-tone method to measure the phase gradients of SFOAEs in high frequency-resolution, and to demonstrate the role of using phase gradient in combination with conventional amplitude spectrum to obtain more reliable results of auditory health screening in the clinic.

## Materials and methods

### Subjects

Sixteen subjects were recruited from Shenzhen Institutes of Advanced Technology, with ages from 23 to 36 years old. All subjects declared that they had no congenital auditory disease in the family and no history of ontological surgery or ototoxic drug usage. A conventional audiogram test was also performed on each subject at standard frequencies (1, 2, 3, 4, 6, and 8 kHz) prior to the experiment. Twelve subjects demonstrated normal hearing with thresholds better than 20 dB HL across all frequencies, and the other four subjects had mild hearing loss over certain frequencies due to long-term exposure to loud sounds. The subjects were told to lie comfortably on a foam-covered bed in a double-walled sound booth during the experimental tests. All subjects gave informed consent and provided permission of their data for scientific purposes. The protocol of this study was approved by the Institutional Review Board of Shenzhen Institutes of Advanced Technology (SIAT- IRB-130124-H0015).

### Equipment

The presentation of the stimuli and the recording of the acoustic response were controlled by a custom Windows PC program implemented in Matlab environment (Mathworks Inc., USA). Signal Processing Toolbox in Matlab was adopted in this manuscript to further analyze the signals. The digital waveforms of the stimuli were initially synthesized from the PC and then sent to an USB sound card (E-MU 0204, Creative Technology Ltd.) with very low noise background and rather low nonlinear distortion *via* a universal ASIO driver. The sound card is a full duplex with two input channels and four output channels, with all channels of pristine resolutions of 24 bits and very high signal-to-noise ratios (SNRs) up to 117 dB. Two ER-2A earphones (Etymotic Research) were connected to the sound card and converted the digital voltage to acoustic sounds to stimulate the auditory system at the ear canal. The acoustic response was simultaneously recorded by an ER-10+ low-noise microphone (Etymotic Research) seated together with the two earphones inside a foam earplug, and digitized by the USB sound card at a sample rate of 48 kHz. All the original raw data were stored for offline analyses.

### Experimental design and procedures

#### Three-interval protocol to extract stimulus frequency otoacoustic emissions

Since SFOAEs share the same frequency as the evoking stimulus, the extraction strategy becomes more complicated than the simple spectral analysis of TEOAEs or DPOAEs (Kalluri and Shera, 2007). A three-interval protocol (Keefe, 1998; Keefe and Ling, 1998; Chen et al., 2013; Jun et al., 2014) based on the two-tone suppression phenomenon (Kemp and Chum, 1980) was used to extract the SFOAEs in the experiment.

**FIGURE 1:**
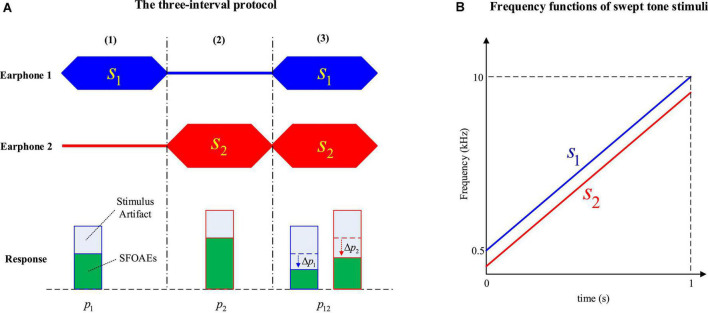
The three-interval protocol to measure stimulus frequency otoacoustic emissions (SFOAEs) **(A)** and frequency functions of swept-tone stimuli **(B)**.

In the three-interval protocol, two stimulus tones (*s*_*1*_ and *s*_*2*_) were presented in a specially designed order within three intervals of equal duration (Figure 1A). When either stimulus (*s*_*1*_ or *s*_2_) was presented alone in the first or the second interval, the acoustic response (*p*_*1*_ or *p*_2_) contained both the stimulus artifact and the evoked SFOAEs. However, when the same stimuli *s*_*1*_ and *s*_*2*_ were presented simultaneously during the third interval, the SFOAEs evoked by either stimulus in the response *p*_12_ would be suppressed by the other stimulus and the amplitude would decrease by △*p*_1_ or △*p*_2_ as a result. In contrast, the stimulus artifacts related with *s*_*1*_ and *s*_*2*_ remained unchanged. When subtracting the response of *p*_*12*_ from *p*_1_*p*_2_:


(1) $$△\,p=p_{1}+p_{2}-p_{12}$$

Most of the stimulus artifacts would be canceled out, and only the SFOAE amplitude changes in the third interval (△*p*_1_ and △*p*_2_), as well as other background noises and interferences, would be left in the residue △*p*.

#### Stimulus generation and presentation

In this study, both stimuli *s*_*1*_ and *s*_*2*_ were swept tones with time-varying frequencies to improve the efficiency of SFOAE measurements (Figure 1B). The swept tones were constructed by customizing the amplitude and phase spectra in the frequency domain, and converting to the time domain to get the temporal waveform *via* an inverse fast Fourier transform (iFFT) (Müller and Paulo, 2001; Chen et al., 2013).

In the experiment, the duration of all three intervals was kept at 1 s. The frequency of *s*_*1*_ (the probe tone) was increased linearly from 0.5 to 10 kHz within 1 s, and the frequency of *s*_*2*_ (the suppressor tone) was kept 200 Hz lower than *s*_*1*_ (Figure 1B). The level of *s*_*1*_ (*L*_*1*_) was increased from 45 to 60 dB FPL at a 5-dB step, and the level of remained constant at 80 dB FPL.

#### Experimental procedures

During the experiment, a foam earplug of the selected size was carefully inserted into the ear canal of the subject. Then the digitally generated swept tones were presented by the two earphones in a three-interval fashion as shown in Figure 1, and the acoustic response at the ear canal was simultaneously collected by the microphone seated inside the earplug. For each signal condition, the same stimulus tones were repeatedly presented for 20 times and the responses were digitally averaged to improve the SNR. In the experiments, the stimulus level was equalized across frequencies according to the forward sound pressure (Chen et al., 2014) to avoid the impacts of standing waves. As a comparison, another session with no stimulus level calibration, during which there might be excessive stimulus level within a certain frequency range, was also carried out to examine the effects on the swept-tone SFOAEs.

For verification purposes, the earplug was also inserted into a plastic uniform tube with one end closed and the same procedures were performed to measure the response. The tube was 25 mm in length and 7 mm in diameter, approximately the same size as an adult ear canal. The acoustic response was recorded and analyzed to compare the differences between the human ear and the plastic tube.

#### Data analysis

A tracking filter that can dynamically follow the instantaneous frequency of the target was used to extract the swept-tone SFOAEs in this study, and the details of the swept-tone and tracking filter could be found in Abdala et al. (2018) and Liu et al. (2020). Another advantage of the tracking filter is that it can easily attenuate the unwanted signal components by placing zeros around the corresponding frequency (Chen et al., 2013). As noted in Eq. 1, two major swept-tone SFOAE components (△*p*_1_ and △*p*_2_) coexisted in the residue response △*p*. Since nonlinear system distortions might be involved at high stimulus level (80 dB FPL) for *s*_*2*_, only the swept-tone SFOAEs evoked by *s*_*1*_ (the △*p*_1_ component) were analyzed in this study. Accordingly, there was one pole to track the △*p*_1_ component, and one zero to attenuate the △*p*_2_ component in the setup of the tracking filter. The signal passed through the tracking filter four times to improve the filter performance (Chen et al., 2013).

**FIGURE 2:**
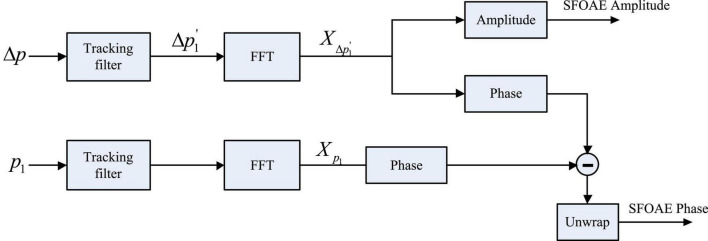
Signal processing procedures to get the amplitude and phase of swept-tone stimulus frequency otoacoustic emissions (SFOAEs). The phase subtraction in the last step was to cancel the phase shifts introduced by the stimulus and the tracking filter.

As shown in Figure 2, the tracking filter was applied to the temporal waveform of △*p* to get a dynamic estimate (△*p*_1_) of the swept-tone SFOAE component △*p*_1_. Then a fast Fourier transform (FFT), with a fixed 1-s length Hanning window, was performed on the △*p*_1_, and the magnitude of the FFT result *X*_△*p*1′_ was taken as the amplitude spectrum of the swept-tone SFOAEs. Meanwhile, the center frequency of the tracking filter was set 100 Hz above the frequency of △*p*_1_, and the amplitude of the filter output was calculated as the reference of the noise floor.

To get the phase of the swept-tone SFOAEs, the same tracking filter that was used to obtain △_*p*1_′ was applied to the response of *p*_*1*_ in Figure 1, and an FFT was performed on the corresponding filter output to get the spectral complex *X*_*p*1_ (Figure 2). Then the phase of *X*_*p*1_ was subtracted from the phase of *X*_△*p*1′_ (FFT result of △_*p*1_′), and the unwrapped phase difference was calculated as the phase of the swept-tone SFOAEs. The phase subtraction was used to eliminate the delays introduced by the recording system and the tracking filter, so that the phase gradient attribute to the swept-tone SFOAEs could be truly revealed.

Since the frequency of the stimulus continuously changed with time, the evoked swept-tone SFOAEs were also continuous in frequency, making it possible to obtain the phase spectrum of SFOAEs in high definition to avoid possible discontinuities. For an SFOAE phase spectrum ϕ(*f*) prepared by the procedures in Figure 2, the group delay τ, defined as the transit time of a signal through a system, could be calculated by:


(2) $$\tau =-\frac{1}{2\,\pi }\,\frac{d\,\phi \,f}{d\,f}$$

The group delay of SFOAEs was a rather useful measure of the travel time of the OAE signals inside the cochlea, and it could provide quite useful information about the functional status of the OHCs and the sharp tuning of the cochlea (Shera and Guinan, 2003; Siegel et al., 2005). In this study, the group delays were calculated at discrete frequencies (*f*_*i*_) from 1 to 10 kHz (at a 1-kHz step) for all the subjects. For each discrete frequency *f*_*i*_, the phase-frequency function from *f*_*i*_−100*Hz* to *f*_*i*_ + 100*Hz* was fitted with a straight line and the slope was used to calculate the group delay according to Eq. 2.

## Results

### Swept-tone stimulus frequency otoacoustic emissions in subjects with normal hearing

**FIGURE 3:**
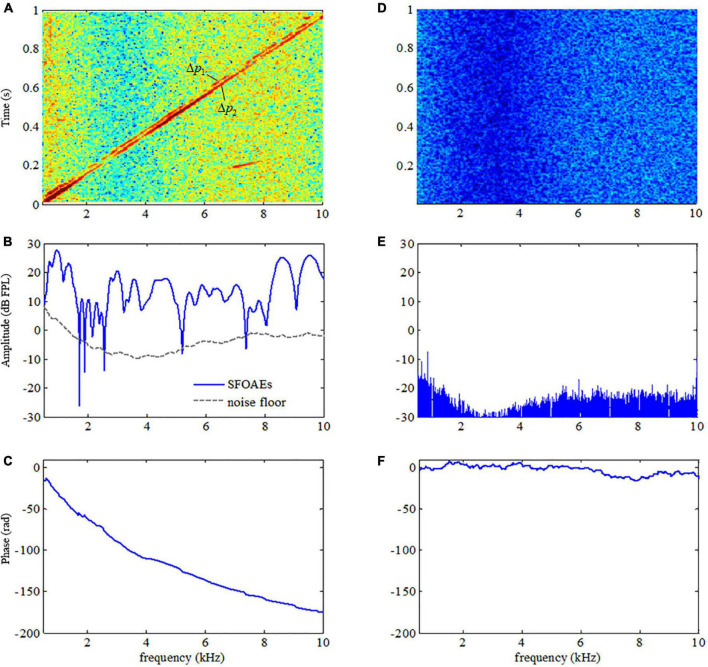
Difference in the spectrograms, amplitude, and phase spectra between the acoustic responses in a human ear (**A–C**, respectively) and in a plastic tube (**D–F**, respectively).

The presence of OAE signals is a distinctive feature of the healthy human ear that makes it different from other passive systems such as acoustic tubes. The use of swept tones made it possible to observe the OAE features in such a high definition. For comparison purposes, swept-tone SFOAEs were measured in both human ears with normal hearing and a plastic tube of similar sizes under the same signal conditions (*L*_1_ = 50 dB FPL) in this study. A typical comparison of the spectrogram (energy distribution as a function of time and frequency), amplitude and phase spectra between the two responses (represented by △p in Eq. 1) was shown in Figure 3. The most important finding was that the responses in the human ear and plastic tube showed dramatically different patterns. For the response in the human ear, two ascending lines, which reflected the energy concentrations in the residue response, were clearly observed in the spectrogram (Figure 3A). The two lines corresponded to the two SFOAE components (△*p*_1_ and △*p*_2_) and had quite similar frequency patterns as their evoking stimuli (Figure 1B). Then a tracking filter was applied to extract the △*p*_1_ component, and the filtered amplitude and phase spectra were shown in Figures 3B,C, respectively. The SFOAE amplitude in Figure 3B consisted of slow baseline variations and rapid spectral periodicity (or fine structures) indicated by alternating peaks and troughs. The overall SFOAE amplitude could get around 30 dB FPL above the noise floor for the subject with normal hearing. The pattern of the fine structures is unique for each specific subject. Another unique feature of the ear response was that the phase decreased dramatically as the frequency increased: the amount of the phase decrease could exceed 150 rad when the frequency increased from 0.5 to 10 kHz. However, for the response in the plastic tube, no swept-tone SFOAEs were observed in either the spectrogram (Figure 3D) or the amplitude spectrum (Figure 3E). The phase no longer demonstrated steep gradient and fluctuated around 0 as the frequency increased (Figure 3F).

### Swept-tone stimulus frequency otoacoustic emissions in subjects with mild hearing loss

**FIGURE 4:**
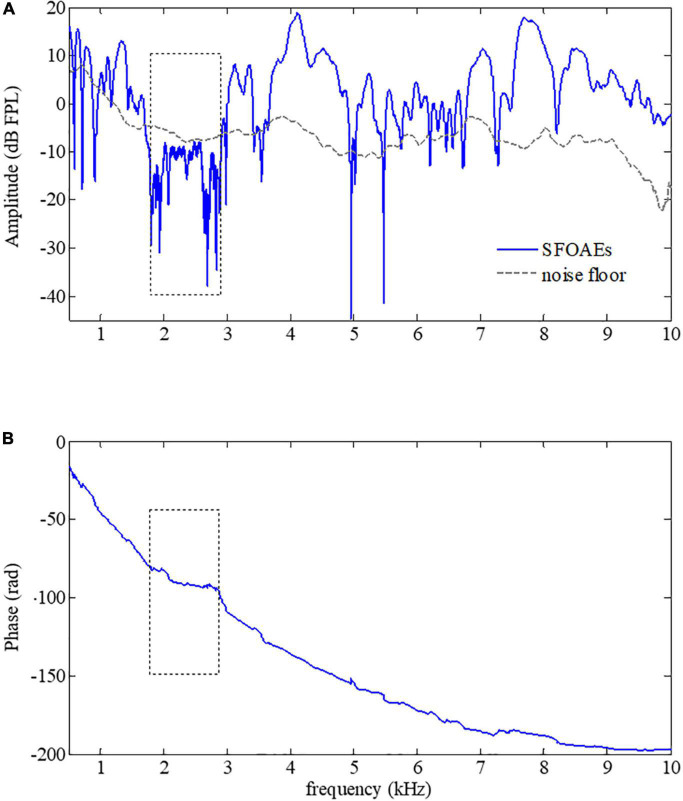
Amplitude **(A)** and phase **(B)** of swept-tone stimulus frequency otoacoustic emissions (SFOAEs) of a subject with hearing loss from 2 to 3 kHz (indicated by dotted boxes).

Since SFOAEs are closely related to the normal functions of the cochlea, any alterations in the cochlear functions accompanied by auditory functional abnormality would result in changes in SFOAEs. A typical example of the swept-tone SFOAEs of a subject with mild hearing loss of 2–3 kHz was shown in Figure 4. As shown in Figure 4A, the overall baseline amplitude of the SFOAEs fell below the noise floor within 2–3 kHz, which was consistent with the frequency region of the hearing loss. However, there was a large amplitude of SFOAEs over other frequencies. For the phase spectrum in Figure 4B, although steep phase gradients could be observed at most frequencies, the phase function became rather flat when the frequency was from 2 to 3 kHz. The flattening of the phase was consistent with the SFOAE amplitude reduction, as well as the region of the hearing loss. The abrupt phase discontinuities at other frequencies (such as 5–7.3 kHz) were due to the lower SNR at the trough of the SFOAE fine structures where the phase estimation was more susceptible to random noises.

### Phase gradients of swept-tone stimulus frequency otoacoustic emissions with artifacts

**FIGURE 5:**
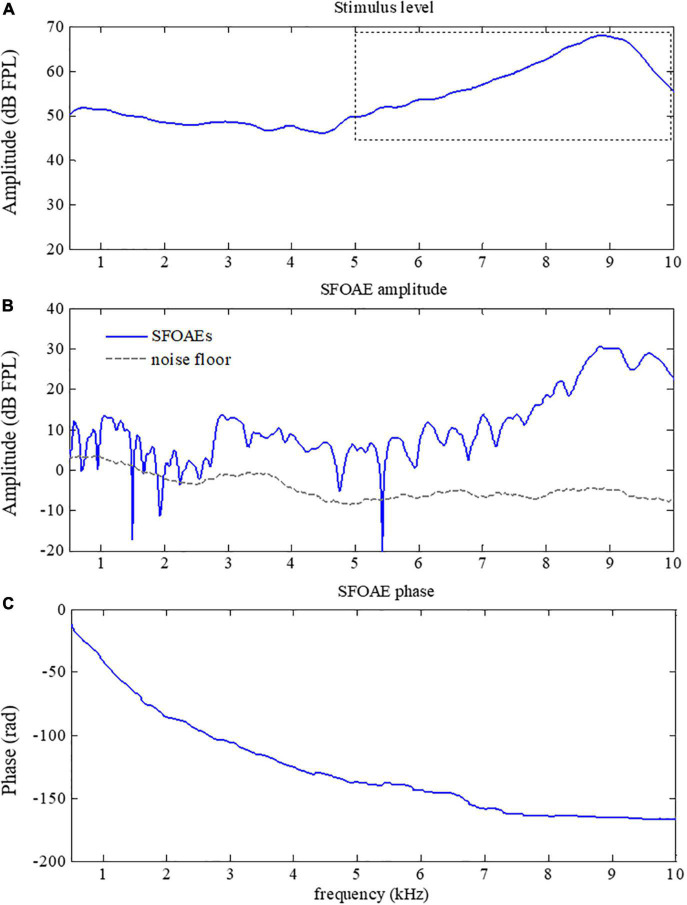
Amplitude **(B)** and phase **(C)** of swept-tone stimulus frequency otoacoustic emissions (SFOAEs) with artifacts above 5 kHz caused by excessive stimulus level (indicated by the dotted box in **A**).

The SFOAEs of different frequencies originate from activities of OHCs at different positions along the cochlea. In practice, equalization of stimulus level across frequencies was usually desired so that SFOAEs from different frequencies could be compared. However, it is very common to have excessive stimulus level over certain frequency ranges without calibrations of stimulus levels, leading to large artifacts that could not be ignored in the SFOAE analyses (a typical example was shown in Figure 5). As shown in Figure 5B, a large amplitude of SFOAEs could be observed across all frequencies from 0.5 to 10 kHz, and it could reach up to 25 dB FPL around the frequency of 9 kHz. However, as we checked the corresponding stimulus level in Figure 5A, it was found that the stimulus level above 5 kHz was much higher than the expected level of 50 dB FPL. Such excessive stimulus level (as high as 20 dB) could introduce nonlinear system distortions that could not be canceled out during the subtraction in Eq. 1 or the filtering by the tracking filter, resulting in unexpected artifacts during the SFOAE measurements (Whitehead et al., 1995; McCreery et al., 2009; Burke et al., 2010; Chen et al., 2014). Fortunately, such artifacts could be correctly identified by the abnormal phase gradient in Figure 5C, where the phase gradually violated the steep gradient pattern of OAE signals above 5 kHz. The flattening of the phase curve suggested that there might be a large amplitude of system distortion involved, leading to unreliable results in the extracted signals above 5 kHz.

### Groups delays of swept-tone stimulus frequency otoacoustic emissions

**FIGURE 6:**
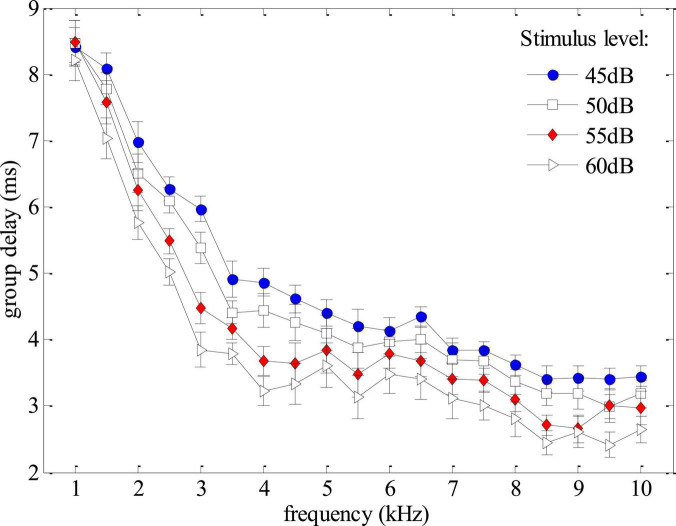
Group delays averaged across all subjects as functions of frequency and stimulus level derived from phase gradients of the swept-tone stimulus frequency otoacoustic emissions (SFOAEs).

Group delays, obtained from the derivative of the phase versus frequency function (Eq. 2), could provide a non-invasive tool to monitor the cochlear tuning that is important for cochlear healthiness (Shera and Bergevin, 2012) as well as the frequency selectivity which is essential for speech perception (Evans, 1975). In this study, the SFOAEs were measured using swept tones to provide SFOAE phases in high frequency-resolution, making it possible to obtain reliable SFOAE group delays with high efficiency. In the experiment, the group delays of the swept-tone SFOAEs were measured at 10 discrete frequencies (from 1 to 10 kHz at a step of 1 kHz) under different stimulus levels. The means and standard deviations of the group delays averaged across the 12 normal-hearing subjects were shown in Figure 6. For the stimulus of 45 dB FPL, the average group delay decreased from around 8.5 ms to about 3 ms as the frequency increased from 1 to 10 kHz. The group delay decreased more rapidly at lower frequencies. When the stimulus level increased from 45 to 60 dB FPL, the group delay at a given frequency decreased monotonously, which was consistent with the findings of relevant studies (Shera and Guinan, 2003; Bentsen et al., 2011; Shera and Bergevin, 2012). Due to the impacts of low-frequency noises in the ear canal, the group delays of different stimulus levels almost overlapped at 1 kHz. Since the phase versus frequency function became rather flat as consequence of hearing loss (Figure 4), the estimated group delays within the corresponding frequency range would approach 0 ms and the group delay pattern would be quite different from Figure 6 for subjects with hearing loss.

## Discussion

### Usefulness of stimulus frequency otoacoustic emission phase gradients in auditory health screening

The study showed that the phase gradients of swept-tone SFOAEs were rather useful to help improve the reliability of auditory health screening using OAE measurements. As observed in Figure 3, the steep phase gradient of OAE signals is a unique feature of the normal functioning human cochlea. Such steep phase gradient would totally disappear if measurements were made in a passive tube (Figure 3) or partially vanish if auditory functional abnormality like hearing loss existed within certain frequency ranges (Figure 4). The close relation between the OAE phase gradients and cochlea healthiness is also reported in relevant studies using fixed-frequency tones (Martin et al., 2009; Avan et al., 2011; Shera and Bergevin, 2012). However, the phase information of OAEs is habitually abandoned due to insufficient frequency-resolution in clinical applications. Currently, only the results of OAE amplitude vs. noise level were provided and the presence of OAEs is determined by whether the OAE amplitude is above the noise level (Kemp et al., 1990; Erenberg et al., 1999; Abujamra et al., 2013). One problem is that the screening results are largely dependent on the reliability of the noise estimate. Since there is no universal standard for noise estimation, different levels of noise might be obtained if different algorithms are used for the noise calculation (Attias et al., 2001; Reavis et al., 2008), leading to inconsistent results among different methods. In this study, the noise floor was obtained by setting the center frequency of the tracking filter 100 Hz above the swept-tone SFOAEs (Figures 3–5). However, slight differences in the estimated noise floor might be expected if the tracking filter was set in different ways, which might result in differences in the identification of a possible hearing-loss region in Figure 4A. In contrast, the calculation of SFOAE phases is independent of the noise estimation, making it more suitable and reliable to indicate the presence of OAE signals or the existence of possible auditory healthy issues (hearing loss in this study) over certain frequencies (Figure 4B). However, it is recommended that the phase of SFOAEs should be used in combination with the conventional amplitude spectra for more accurate auditory health screening in clinical practices. Moreover, the group delays calculated from the phase gradient could also be used to detect the abnormality of cochlear tuning that demonstrates evident alterations at the early stage of auditory functional disorders such as hearing loss (Francis and Guinan, 2010; Shera and Bergevin, 2012).

### Phase gradients and group delays to detect otoacoustic emission artifacts

The present study also showed that the SFOAE phase gradients could help to identify possible artifacts that otherwise could be falsely treated as actual OAE signals. As mentioned earlier, the major difference between OAE signals and other responses is that the unique phase gradient (Figure 3) is so steep that the derived group delay is about 8.5 ms at 1 kHz for all subjects (Figure 6), not including the system round-trip delay that the stimulus spending on traveling along the outer and middle ears. The steep phase gradient, as well as the group delay, originates from the signal front delay which is the time difference between the onset of the basilar membrane (BM) and stapes, and the filter delay that the BM spends on building the peak of the traveling wave (Ruggero, 2004). The filter delay of the BM, the major portion of the OAE group delay, is a unique physiological parameter closely related to the frequency selectivity of the cochlear tuning, with sharper tuning corresponding to longer group delays (Shera and Guinan, 2003; Ren et al., 2006). If there is no cochlear tuning involved (such as the response in a passive tube), the steep phase gradient would disappear (Figure 3) and the group delay would approach 0 as a consequence. In Figure 5, system distortions were involved above 5 kHz due to the excessive stimulus level, leading to incomplete cancellation of stimulus artifacts during the subtraction in Eq. 1. The remaining stimulus artifacts with zero group delay would dominate the low-level SFOAE components (△*p*_1_ and △*p*_2_) in the residual response △p (Eq. 1), resulting in the violation of steep phase gradients at the corresponding frequency range (Figure 5C). However, it would be mistaken if we determined the presence of large OAE signals by merely checking the high SNR in the amplitude spectrum above 5 kHz (Figure 5B). Therefore, checking the steep phase gradient in combination with the amplitude spectrum is a more reliable way to distinguish OAE signals from other irrelevant interferences or unexpected noises.

## Conclusion

In this paper, SFOAEs were measured with swept tones in high frequency-resolution, so that the phase spectrum that is conventionally not feasible in auditory research or clinical tests could be obtained in a quite efficient way. The results demonstrated that the SFOAE phases in human ears showed steep gradients as the frequency increased, and such steep gradients are unique features that make the human-ear response different from other passive systems. The steep phase gradients could help to efficiently validate frequency regions of auditory functional abnormality, and to identify stimulus artifacts that could be mistakenly treated as evident OAE signals in practical applications. The pattern of the group delays derived from SFOAE phase gradients might be used to reflect the cochlear latency characteristics that were useful to evaluate the sharpness of the cochlear tuning and the normalcy of the cochlear frequency selectivity. The study suggested that using swept tones to measure SFOAEs and involving the phase information in combination with the amplitudes could be a rather promising approach to help improve the reliability of current hearing screening in the clinic.

## Data availability statement

The datasets presented in this article are available on reasonable request from the corresponding authors. Requests to access the datasets should be directed to SC.

## Ethics statement

The protocol of this study was approved by the Institutional Review Board of Shenzhen Institutes of Advanced Technology, Chinese Academy of Sciences (SIAT- IRB-130124-H0015). The patients/participants provided their written informed consent to participate in this study.

## Author contributions

SC and YT: conceptualization. XW and MZ: data curation. SC, GL, and MZ: funding acquisition. XW and SC: methodology, writing—original draft, and writing—review and editing. SC: supervision. XW, YH, and ZL: validation. XW, XH, and HP: visualization. All authors contributed to the article and approved the submitted version.
